# Evaluating different scoring algorithms to assess communication ability for individuals living with Angelman syndrome: the Observer-Reported Communication Ability (ORCA) measure

**DOI:** 10.1186/s41687-026-01072-7

**Published:** 2026-05-02

**Authors:** Bryce B. Reeve, Li Lin, Abigail Rader, Nicole Lucas, Kelly L. Gordon, Molly McFatrich, Allyson Berent, Harrison N. Jones

**Affiliations:** 1 Duke University Health System, Durham, USA; 2 Foundation for Angelman Syndrome Therapeutics, Austin, USA; 3 East Carolina University, Greenville, USA

**Keywords:** Clinical outcome assessment, Communication, Rare disease, Psychometrics

## Abstract

**Background:**

Many neurodevelopmental disorders are associated with significantly impaired communication that impacts the individuals and their families’ daily activities. The objective of this study is to evaluate the effectiveness of multiple scoring algorithms for the Observer-Reported Communication Ability (ORCA) measure in assessing communication abilities in individuals living with Angelman syndrome and other neurodevelopmental disorders with severe language impairments.

**Methods:**

This secondary data analysis used caregiver-reported data from the ORCA validation study (*n* = 249) and the Angelman Syndrome Natural History Study (*n* = 165). Performance of the scoring algorithms was evaluated based on item response theory information functions, the percentage of scores at the floor and ceiling, and the Pearson correlation of scores between the ORCA measure and the Communication and Symbolic Behaviors Scale (CSBS).

**Results:**

The ORCA measure’s scoring algorithms, which include both emerging and mastery levels of communication behaviors (“Emerging & Mastery”), captured lower levels of ability with greater precision than the algorithm that only assesses mastery levels of behaviors. For overall communication ability in the validation study, the Emerging & Mastery algorithm had floor and ceiling effects of 0.4% each, while the Mastery algorithm had effects of 1.2% and 0.0%, respectively. Both ORCA scoring algorithms for overall communication had strong correlations with the CSBS (*r* = 0.85 and 0.83, respectively). Similar results were found when scoring ability at the expressive, receptive, and pragmatic (social) communication levels.

**Conclusions:**

Selection of the appropriate ORCA scoring algorithm for use in research studies should be based on the communication concept of interest for the intervention being evaluated and the characteristics of the neurodevelopmental disorder population being assessed. This study reinforces the need for robust assessment tools that can adapt to the unique challenges of rare disease populations.

## Introduction

Recent advances in gene-targeted therapies have led to an escalation of clinical trials to evaluate their safety and efficacy within a range of neurodevelopmental disorders, including individuals living with Angelman syndrome (AS) [1]. AS is a rare, neurogenetic disorder caused by loss of function of the maternally inherited allele of the *UBE3A* gene [2–4]. AS is associated with impaired motor function, ataxia, hypotonia, epilepsy, impaired cognitive and executive functioning, sleep disturbance, and severe communication impairments characterized by minimal to no verbal speech production and expressive and receptive language impairments [5–9]. These limitations have an impact on daily activities and social, family, and school role functioning [10]. Family caregivers report that communication is one of the most important outcomes they would like to see improved in their children as an indicator of treatment efficacy in clinical trials [11].

While several measures of communication ability exist [12], these measures are limited in one or more ways that make them less appropriate for clinical trials for individuals living with AS and other neurodevelopmental disorders (NDDs) with similar impacts on communication. Importantly, many communication measures were designed for verbal populations, whereas individuals living with AS are primarily nonverbal and use alternative modalities to communicate, such as gestures, signs, vocalizations, and other forms of augmentative and alternative communication devices [7, 13–18]. Measures that do not assess these alternative communication modalities can introduce floor effects and fail to capture meaningful changes in communication ability. Additionally, some communication measures include performance-based tasks, which can be challenging for individuals living with AS with dyspraxia or apraxia, where the individual performs the task in an unnatural clinical setting and in front of an administrator with whom they are not familiar. Lastly, many communication measures were designed to guide therapeutic goals by special educators or speech-language pathologists and were not intended for use in clinical trials to assess change over time.

The Observer-Reported Communication Ability (ORCA) measure was designed specifically for the AS population and includes communication concepts and associated behaviors that are relevant from the perspectives of communication experts and family caregivers [19–22]. The questionnaire is completed independently by the family caregiver who has spent the most time observing the child’s communication abilities. The ORCA measure was specifically designed for use in clinical trials to evaluate communication ability pre- and post-therapeutic intervention. The development team followed best practice recommendations for designing clinical outcome assessments and directly included parent advocates both in the project oversight and evaluation of the measure [23–26].

The current scoring algorithm for the ORCA measure reflects two key aspects of communication ability. First, it provides a single overall score that incorporates the individual’s expressive, receptive, and pragmatic (i.e., social) forms of communication. The rationale for an overall score includes (1) parsimony—the need to have a single comprehensive score that could be used in a trial endpoint to include all aspects of communication relevant to the AS population, and (2) empirical data collected from caregivers of individuals with AS that supports a dominant single overall construct of communication ability [19, 27]. The second key aspect is that the score reflects an individual’s ability to “master” specific communication concepts like requesting more of something, making choices, and greeting someone. “Mastery” is the extent to which the caregiver reports that their child performed a communication behavior “almost all the time” over the past 30 days on items in the ORCA measure. This approach was initially selected to demonstrate in clinical trials those behaviors an individual demonstrated consistently and not infrequently.

Since the development and implementation of the ORCA measure in research studies among individuals living with AS and with other NDDs, feedback from end users has informed the design of alternative scoring algorithms. Feedback included the need to capture emerging behaviors that may be starting but not yet a routine aspect of their communication, and the desire to have separate expressive, receptive, and pragmatic communication scores to reflect that individuals may have stronger abilities or growth in one but not consistently across all areas. Accordingly, the present secondary data analysis aims to (1) evaluate alternative algorithms that generate separate scores for expressive, receptive, and pragmatic communication, incorporating emerging behaviors, and (2) guide end users on the strengths and limitations of each scoring approach.

## Methods

### Participants

This secondary data analysis incorporates data from two studies that included family caregivers of individuals living with AS. The first dataset comes from the original validation study for the ORCA measure involving 249 caregivers, and the second dataset was obtained from the Angelman Syndrome Natural History Study involving 165 caregivers. The datasets were not merged because many caregivers likely participated in both studies, given that AS is a rare disease, and participants are not identified. Institutional approval for this study was obtained from Duke University Institutional Review Board.

The original validation study enrolled caregivers 18 years of age or older, who were able to read and understand English, resided in the United States, had access to an internet-enabled device, and lived with a child with AS. The child had to be between 2 and 40 years of age and have molecular confirmation of their diagnosis. A study recruitment flyer was posted on the Foundation for Angelman Syndrome Therapeutics (FAST) Facebook page and circulated within relevant forums. Caregivers completed the ORCA measure through an online REDCap survey. Additional details are provided elsewhere [19].

The Angelman Syndrome Natural History Study (AS-NHS) is a longitudinal multicenter study enrolling individuals with a molecular diagnosis of AS between birth and 60 years old. Participants were enrolled at one of 10 study sites: Boston Children’s Hospital, Rady Children’s Hospital San Diego, Alberta Children’s Hospital, British Columbia Children’s Hospital, Children’s Hospital of Eastern Ontario, Rush University Medical Center, Emory University Hospital, University of North Carolina Medical Center, Monroe Carell Jr. Children’s Hospital at Vanderbilt, and Children’s Hospital Colorado. The ORCA measure was completed by adult family caregivers either by a paper form or an online portal. More details are provided elsewhere [28].

### Measures

#### ORCA measure

The ORCA measure includes 81 total items and, on average, takes about 15 min for caregivers to complete. Seventy items inquire about observable communication behaviors within 23 (or 24, depending on the selected algorithm) concepts that reflect expressive, receptive, and pragmatic forms of communication (Table 1). Caregivers indicate if their child has exhibited the behavior in the past 30 days, and the response options include “no or only once,” “sometimes,” or “yes, almost all the time.” Eleven additional items capture information about the individual’s unique ways of communicating, including the modalities they use, their current vocabulary, and aspects of language complexity such as how many words, symbols, and gestures are used to communicate a single message.

**Table 1:** Concepts contributing to estimating communication ability scores on the ORCA measure by form of communication

Expressive Communication	Receptive Communication	Pragmatic Communication
Seek Attention	Respond to Name	Greeting
Direct Attention	Understand Mood	Comfort Others
Refuse Object	Understand Isolated Words	Play Games
Request Object	Turns in Conversation	Use Names (others & self)
Request Object Out of View	Make Choices	
Request “More”	Respond to Familiar Directions	
Communicate Understanding	Respond to New Directions	
Asking Questions	Answer Questions	
Communicate with Others		
Telling About the Past*		
**“Vocabulary”**		
Number of verbal words		
Number of symbols on an assistive device		

*The concept “telling about the past” is currently included in the ORCA measure but is not part of the scoring metric at this time. In the initial psychometric study, too few caregivers of individuals with AS endorsed this concept for the investigators to feel confident in the stability of the IRT item parameters. This concept represents very high communication ability whose importance was supported by the concept of elicitation interviews and cognitive testing. Thus, it was not removed. However, more data will need to be collected on the concept before integrating it into scoring

Item response theory (IRT) is used as the measurement model to calibrate the scale and to estimate an individual’s ORCA score [29, 30]. The IRT model is fit to variables that represent communication concepts (Table 1) and not to individual questions on the ORCA measure. This is because multiple ORCA questions may be asked about a single communication concept. For example, Table 2 presents two questions (10a and 10b) that both reflect the communication concept of “Asking Questions.” Fitting the IRT model for each question results in the pair of example questions being locally dependent—a violation of a key assumption (i.e., locally independent items) of the IRT model used in this study. Thus, the two example questions are combined into a single variable (unit of analysis) that is scored 0, 1, or 2. This process of removing local dependence is equivalent to the methodology of designing “testlets” in educational testing; for example, when there are related questions within a reading comprehension passage of a standardized exam [31].

**Table 2:** Creating the “Asking Questions” variable from two questions on the ORCA measure with the Mastery and the Emerging & Mastery Scoring algorithms

Questions 10a and 10b on the ORCA Measure for the Caregiver to Answer about their Child					Scoring Algorithms Combining the Two Questions into a Single Variable Scored from 0 to 2	
Instructions: Please tell us how your child requested more of something. In the past 30 days,					**“Mastery” Scoring Algorithm**	**“Emerging & Mastery” Scoring Algorithm**
#	Question Stem	Response options				
					**Score = 0** if caregiver doesn’t answer “Yes, almost all the time” to 10a or 10b	**Score = 0** if caregiver doesn’t answer “Sometimes” or “Yes, almost all the time” to 10a or 10b
10a	Did your child use gestures/signs, words/word approximations, or symbols on a device to ask you **simple** questions (example: Where’s mommy? )?	No or only once	Some- times	Yes, almost all the time	**Score = 1** if caregiver answers “Yes, almost all the time” only to 10a.	**Score = 1** if caregiver answers “Sometimes” to 10a and “No” to 10b
						**Score = 2** if caregiver answers “Yes” to 10a and “No” to 10b
10b	Did your child use gestures/signs, words/word approximations, or symbols on a device to ask you **complex** questions (example: Why do I have to brush my teeth? )?	No or only once	Some-times	Yes, almost all the time	**Score = 2** if caregiver answers “Yes, almost all the time” to 10b.	**Score = 3** if caregiver answers “Sometimes” to 10b
						**Score = 4** if caregiver answers “Yes, almost all the time” to 10b

The ORCA “Mastery” scoring algorithm combines items into a testlet, which we refer to as a communication “concept,” based on whether or not the caregiver answers that their child performed a behavior “Yes, almost all the time” in the past 30 days. As seen in the next-to-last column of Table 2 for the example concept of “Asking Questions,” an individual receives a score of 1 if the caregiver answers “Yes, almost all the time” to question 10a (asking simple questions) and receives a score of 2 if the caregiver answers “Yes, almost all the time” to question 10b (asking complex questions). The ORCA Mastery scoring algorithm does not distinguish between the answers of “No or only once” and “Sometimes” but rather focuses on communication behaviors that are consistently exhibited over the past 30 days.

The ORCA “Emerging & Mastery” scoring algorithm was created to distinguish among the three response options at the concept level. As seen in the last column of Table 2, an individual receives a score of 1 if the caregiver answers “Sometimes” to 10a, a score of 2 if the caregiver responds “Yes, almost all the time” to 10a, a score of 3 if the caregiver answers “Sometimes” to 10b, and a score of 4 if the caregiver answers “Yes, almost all the time” to 10b. We worked with communication experts to develop appropriate levels for each communication concept (Table 1) that balanced the hierarchical levels of ability reflecting communication developmental steps, assumptions of the measurement model, and the need for sufficient sample sizes to estimate model parameters for scoring the ORCA measure.

#### Communication and symbolic behaviors scale DP infant-toddler checklist (CSBS)

The CSBS has been validated as a reliable and sensitive screening tool for early communication difficulties in AS and other neurodevelopmental disorders, with strong internal consistency and test–retest reliability, though floor effects limit its sensitivity to small gains in severely affected individuals [12, 32–34].

The CSBS was completed by caregivers in the original validation study but not in the AS-NHS. It includes 24 questions that are summed into a single raw Total score and Communication, Expressive Speech, and Symbolic composite scores, with higher scores representing greater communication ability [35, 36]. The CSBS is used here to compare the strength of associations with the different ORCA scoring algorithms.

### Analyses

We conducted a unidimensional IRT analysis on each ORCA concept. We used Samejima’s graded response model [37, 38], appropriate for ordered categories, to calibrate the scale, including estimation of the discrimination and threshold parameters for each communication concept using marginal maximum likelihood estimation within the original validation dataset. Evaluated model assumptions include unidimensionality of the latent construct (communication ability), local independence between the communication concept variables, and monotonicity of the model form, meaning that higher scores within a concept variable (e.g., “Request More”) reflect higher levels of communication ability. Response pattern IRT scoring uses the Expected A Posteriori (EAP) method [39], and scores are linearly transformed to have a mean of 50 and standard deviation of 10 within the original validation dataset. Thus, we refer to this IRT-based metric as “T-scores”. The IRT parameters are estimated separately for each scoring algorithm (i.e., Mastery, Emerging & Mastery) and for each form of communication (overall, expressive, receptive, pragmatic). Therefore, scores from each calibration cannot be compared against each other as they are not linked, as each form (expressive, receptive, pragmatic) includes different communication concepts. In other words, they exist on different scales and cannot be directly compared. IRT PRO (version 4.2; Scientific Software International, Lincolnwood, IL) was used for IRT analyses.

We examined differences between the two scoring algorithms in three ways. First, we provided IRT scale information functions that display the level of information (precision) for estimating scores conditional on the level of communication ability [29, 40]. Higher information magnitude reflects increased reliability and lower standard error. Information magnitudes of 3.33, 5, and 10 are equivalent to reliability estimates of 0.70, 0.80, and 0.90, respectively. The IRT-based information functions are derived from IRT model parameters obtained in the calibration of the original validation study. There was insufficient sample size to re-estimate the IRT model parameters in the AS-NHS, nor sufficient reason considering that the sample data on the individuals with AS are from the same general population. The IRT models are invariant to subgroups from the same general population [29, 41]. Next, we examined the distribution of scores in both datasets by overall communication ability and expressive, receptive, and pragmatic forms, including floor and ceiling effects. Note that the x-axis for the information functions is the ORCA T-scores; thus, one can see how well the information functions capture communication ability based on where the individuals with AS are located. Third, we compared Pearson correlations between the two ORCA scoring algorithms and CSBS scores, expecting strong correlations (> 0.50; via Cohen, 1992 [42]) with stronger correlations among ORCA T-scores than with the CSBS scores. We expect very strong correlations among the ORCA T-scores as they are pulling information from the same set of questions; the Emerging & Mastery algorithm adds more levels to communication concept variables included in the model.

## Results

### Participants

Demographic data regarding the caregivers (respondents to the survey) and their children with AS are provided in Table 3. The participant characteristics from the original validation study (*n* = 249) and the AS-NHS (*n* = 165) are relatively similar, although there were statistically significant differences in the proportions of the caregivers’ education levels, the children’s ethnicity and racial groups, and the children’s AS genotype.

**Table 3:** Demographics and background information for caregiver participants in the ORCA measure original validation study and the Angelman Syndrome Natural History Study

	Original Validation Study	Natural History Study
	*n* (%)	*n* (%)
	*n* = 249	*n* = 165
**Caregivers (respondents)**		
Age, years (*mean / SD*)	41.9 / 8.6	not available
Ethnicity		
Not Hispanic or Latino	228 (92.3)	not available
Hispanic or Latino	172 (6.9)	not available
Race		
White	227 (91.9)	not available
African-American or Black	3 (1.2)	not available
Asian	8 (3.2)	not available
Native American or Alaskan Native	1 (0.4)	not available
More than one race	4 (1.6)	not available
Relationship to Child		
Mother/Stepmother	220 (88.4)	139 (95.9)
Highest Level of Education*		
High school diploma or less	25 (10.1)	7 (5.8)
Some college/university	58 (23.5)	36 (30.3)
College/University degree	105 (42.5)	44 (37.0)
Graduate degree	59 (23.9)	32 (26.9)
**Individuals living with Angelman Syndrome**		
Age, years (*mean / SD*)	10.6 / 7.9	11.5 / 10.1
Female	112 (45.0)	75 (45.5)
Ethnicity*		
Not Hispanic or Latino	219 (89.8)	132 (80.0)
Hispanic or Latino	22 (9.0)	28 (17.0)
Race*		
White	215 (86.7)	143 (86.7)
African-American or Black	3 (1.2)	14 (8.5)
Asian	5 (2.0)	16 (9.7)
American Indian or Alaska Native		4 (2.4)
More than one race	22 (8.9)	
Other		8 (4.8)
Genotype*		
Deletion Positive	167 (67.1)	88 (53.3)
Mutation/UBE3A	51 (20.5)	44 (26.7)
Imprinting (ICD)	10 (4.0)	10 (6.1)
Uniparental Disomy (UPD)	21 (8.4)	16 (9.7)
Abnormal DNA Methylation		7 (4.2)
ORCA Overall Mastery T-Score (*mean / SD*)	50.0 / 9.6	50.0 / 10.9
ORCA Overall Emerging & Mastery T-Score (*mean / SD*)	50.0 / 9.7	49.3 / 11.6
CSBS Total Score (*mean / SD*)	30.4 / 10.7	not available

Note: * Indicates statistically significant differences (*p*<.05) for the characteristic between the two studies

### Information (precision) of the scoring algorithms

**Fig. 1:**
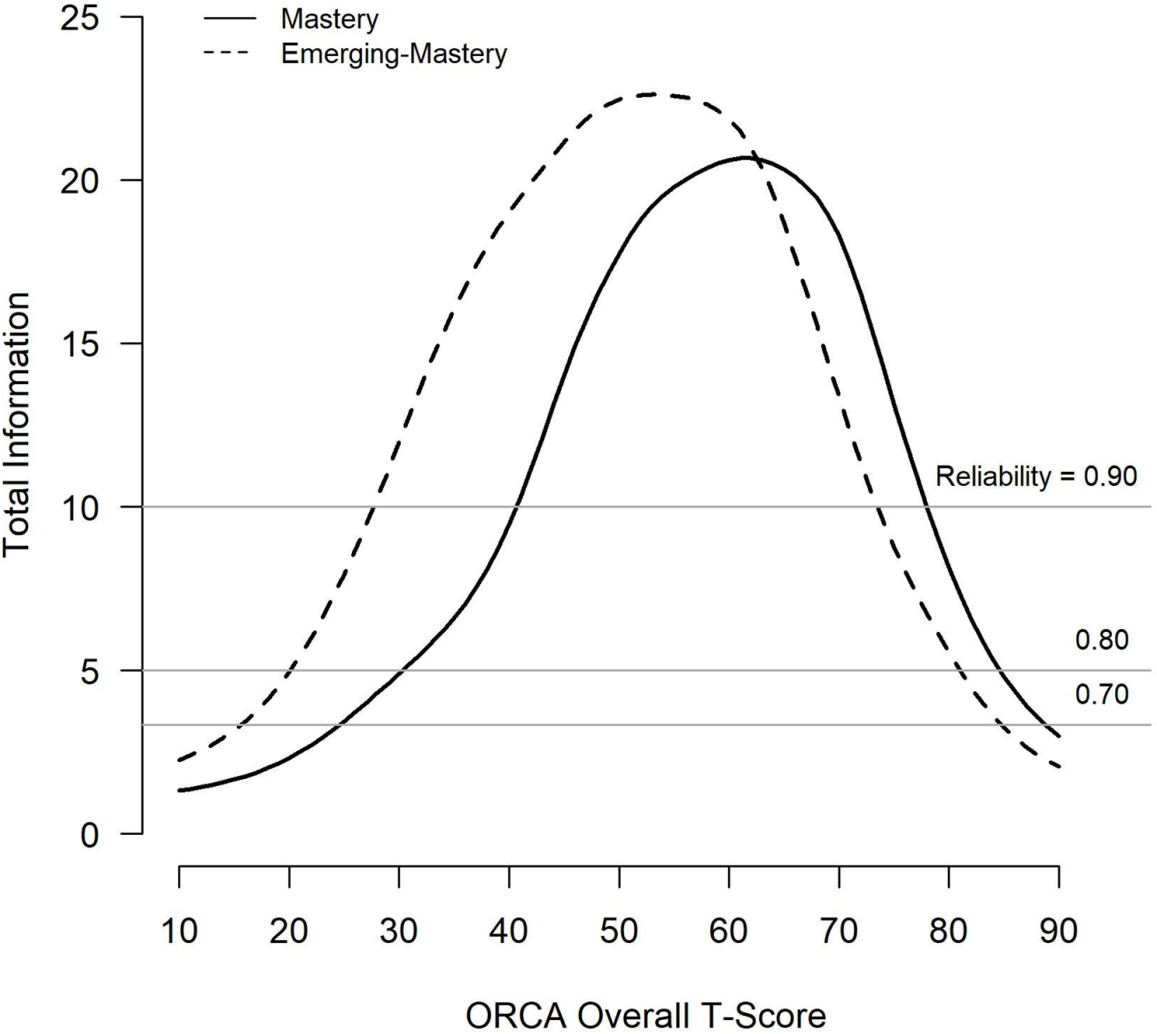
Information functions for the ORCA Measure’s “Mastery” and “Emerging & Mastery” overall communication ability T-scores. The item response theory-based information functions for the “Mastery” level (solid line) and “Emerging & Mastery” level (dashed line) scoring algorithms for the Observer-Reported Communication Ability (ORCA) measure overall communication ability T-scores. More information reflects better precision for estimating ORCA T-scores displayed along the x-axis. Equivalent reliability estimates are provided by horizontal lines for different magnitudes of information

The IRT-based information functions for both the Emerging & Mastery and the Mastery scoring algorithms for the ORCA Overall Communication T-scores are provided in Fig. 1. Using the most stringent reliability threshold of 0.90, the Mastery scoring algorithm’s information function exceeds this threshold for ORCA T-scores from approximately 41 to 78. The Emerging & Mastery scoring algorithm’s information function expands down to an ORCA T-score of 28 and up to approximately 73. If a reliability threshold of 0.70 is used, then precision for ORCA T-scores extends lower to scores of approximately 25 (Mastery) and 16 (Emerging & Mastery).

**Fig. 2:**
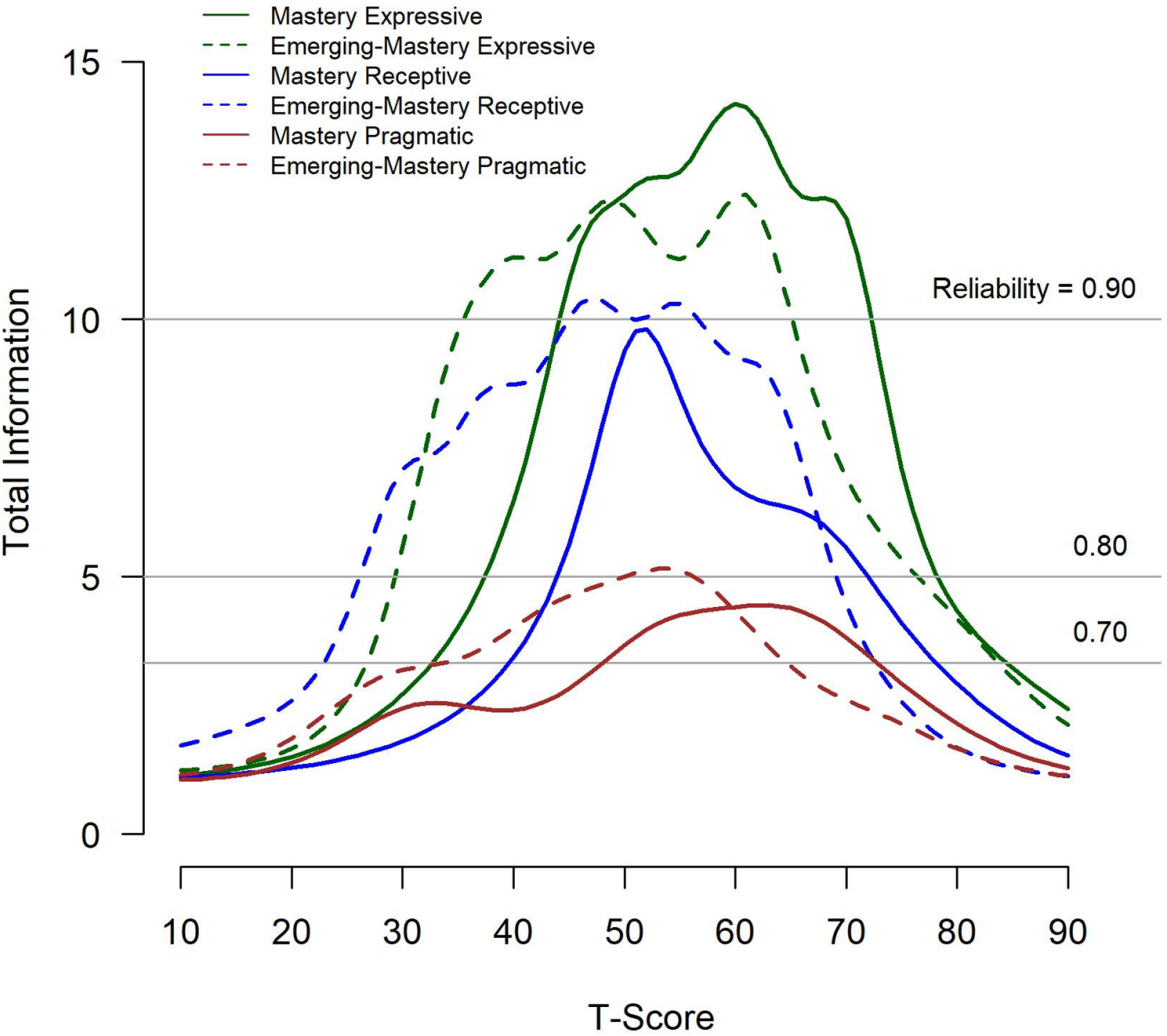
Information Functions for the ORCA Measure’s expressive, receptive, and pragmatic communication ability T-scores. The item response theory-based information functions for the “Mastery” level (solid lines) and “Emerging & Mastery” level (dashed lines) scoring algorithms for the Observer-Reported Communication Ability (ORCA) measure T-scores for expressive communication (green lines), receptive communication (blue lines), and pragmatic communication (red lines). More information reflects better precision for estimating ORCA T-scores displayed along the x-axis. Equivalent reliability estimates are provided by horizontal lines for different magnitudes of information

The information functions for expressive, receptive, and pragmatic forms of communication ability are provided in Fig. 2. The same pattern emerges in that the Emerging & Mastery scoring algorithm extends the precision of estimating ORCA T-scores to the lower end of communication ability scores.

### Distribution of ORCA T-scores in the original validation study and AS-NHS

Table 4 provides descriptive statistics on ORCA T-scores for both scoring algorithms in both datasets. The theoretical possible score ranges shown in the first data column reveal that the Emerging & Mastery scoring algorithms have lower possible scores than the Mastery scoring algorithms, particularly for receptive communication (22.5 and 34.6, respectively). The floor effects are relatively larger for the Mastery scoring algorithms than the Emerging & Mastery scoring algorithms. For both algorithms, there are minimal ceiling effects, with the largest percentage being from Emerging & Mastery Pragmatic Communication in the AS-NHS (4.2%).

**Table 4:** Descriptive statistics for ORCA measure T-score distributions for the Mastery and the Emerging & Mastery scoring algorithms for the original validation study and Angelman Syndrome Natural History Study

	Possible Score Range	Observed Scores								
		Mean	SD	Min	1st Quartile	Median	3rd Quartile	Max	% Floor	% Ceiling
		**Original Validation Study in Angelman Syndrome (** *n* ** = 249)**								
Mastery										
Overall	25.8–83.8	50.0	9.6	25.8	42.8	50.0	56.9	75.9	1.2	0.0
Expressive	31.0–81.5	50.0	9.4	31.0	43.8	49.6	56.4	76.7	2.0	0.0
Receptive	34.6–75.9	50.0	9.0	34.6	42.5	49.6	56.6	73.1	5.2	0.0
Pragmatic	33.6–72.6	50.0	8.4	33.6	42.1	49.1	55.9	72.6	5.2	0.8
Emerging & Mastery										
Overall	18.0–81.2	50.0	9.7	18.0	43.2	50.3	56.8	76.2	0.4	0.4
Expressive	25.9–79.9	50.0	9.5	25.9	43.3	50.2	56.6	72.8	0.4	0.0
Receptive	22.5–71.6	49.9	9.4	22.5	43.1	50.3	57.4	71.6	0.4	1.6
Pragmatic	29.5–68.7	50.0	8.7	29.5	44.0	49.6	56.3	68.7	2.8	2.4
		**Angelman Syndrome Natural History Study (** *n* ** = 165)**								
Mastery										
Overall	*	50.0	10.9	25.8	41.8	49.3	58.0	79.7	1.2	0.6
Expressive	*	50.1	10.5	31.0	43.5	49.5	57.2	78.7	3.6	0.0
Receptive	*	49.1	10.0	34.6	40.9	48.3	57.6	75.9	9.7	1.8
Pragmatic	*	50.6	9.3	33.6	42.1	49.1	56.4	72.6	4.8	1.2
Emerging & Mastery										
Overall	*	49.3	11.6	19.4	41.3	49.0	58.4	80.0	0.0	0.0
Expressive	*	49.6	10.7	25.9	42.2	50.1	57.8	78.4	2.4	0.0
Receptive	*	48.6	11.4	22.5	40.1	48.5	58.5	71.6	0.6	1.8
Pragmatic	*	49.8	9.7	29.5	43.1	49.6	56.4	68.7	1.2	4.2

*The theoretical possible score range is the same for both studies, so the numbers are only provided in the rows for the original validation study. Abbreviations: SD = standard deviation, Min = Minimum observed score, Max = maximum observed score

### Relationship among ORCA T-scores and with the CSBS

Table 5 shows the correlations between the different ORCA T-score forms in both studies, as well as their correlations with the CSBS total and composite scores in the original validation study. Within the ORCA measure, the Mastery and the Emerging & Mastery scoring algorithms show strong correlations within the same communication domains: overall (0.90 and 0.92 within the original validation study and the AS-NHS, respectively), expressive (0.86 and 0.88, respectively), receptive (0.86 and 0.89, respectively), and pragmatic (0.76 and 0.79, respectively). The ORCA measure’s T-scores had similarly strong correlations with the CSBS total and composite scores across the Mastery and the Emerging & Mastery scoring algorithms. The CSBS total scores was correlated at 0.83 with the Mastery overall T-scores and 0.85 with the Emerging & Mastery overall T-scores. The ORCA scores were less related to the CSBS Expressive Speech composite scores, as this CSBS composite focuses on the use of words.

**Table 5:** Pearson correlations among the ORCA measure scoring algorithms and with the Communication and Symbolic Behaviors Scale (CSBS) within the original validation study (*n* = 249) and the Angelman Syndrome Natural History Study (*n* = 165)

	ORCA Measure Mastery Scoring Algorithm				ORCA Measure Emerging & Mastery Scoring Algorithm			
ORCA	Overall	Expressive	Receptive	Pragmatic	Overall	Expressive	Receptive	Pragmatic
Results from the Original ORCA Validation Study								
*Mastery*								
Overall Comm								
Expressive Comm	0.94							
Receptive Comm	0.87	0.71						
Pragmatic Comm	0.85	0.72	0.73					
*Emerging & Mastery*								
Overall Comm	0.90	0.82	0.82	0.79				
Expressive Comm	0.88	0.86	0.73	0.74	0.96			
Receptive Comm	0.83	0.70	0.86	0.73	0.92	0.81		
Pragmatic Comm	0.77	0.66	0.69	0.76	0.89	0.81	0.77	
Results from the Angelman Syndrome Natural History Study								
	**ORCA Measure** **Mastery Scoring Algorithm**				**ORCA Measure** **Emerging & Mastery Scoring Algorithm**			
**ORCA**	**Overall**	**Expressive**	**Receptive**	**Pragmatic**	**Overall**	**Expressive**	**Receptive**	**Pragmatic**
*Mastery*								
Overall Comm								
Expressive Comm	0.95							
Receptive Comm	0.92	0.81						
Pragmatic Comm	0.88	0.77	0.79					
*Emerging & Mastery*								
Overall Comm	0.92	0.85	0.87	0.79				
Expressive Comm	0.91	0.88	0.82	0.77	0.97			
Receptive Comm	0.87	0.78	0.89	0.74	0.95	0.88		
Pragmatic Comm	0.82	0.73	0.76	0.79	0.91	0.85	0.83	
Association of Scores from ORCA Measure with CSBS in Original ORCA Validation Study								
	**ORCA Measure** **Mastery Scoring Algorithm**				**ORCA Measure** **Emerging & Mastery Scoring Algorithm**			
**CSBS**	**Overall**	**Expressive**	**Receptive**	**Pragmatic**	**Overall**	**Expressive**	**Receptive**	**Pragmatic**
Total	0.83	0.74	0.80	0.74	0.85	0.78	0.86	0.75
Communication	0.78	0.71	0.71	0.69	0.77	0.72	0.78	0.67
Expressive Speech	0.52	0.49	0.48	0.44	0.57	0.56	0.52	0.53
Symbolic	0.78	0.64	0.79	0.73	0.79	0.71	0.84	0.71

Note: The Communication and Symbolic Behaviors Scale (CSBS) was only administered in the original validation study

## Discussion

As the ORCA measure continues to be used in research studies with AS and other NDDs, it is critical to ensure that the scores accurately reflect the individual’s communication ability levels. Our study expands on the original scoring algorithm, which estimates a single overall communication score that reflects an individual’s ability to consistently perform communication behaviors over the past 30 days (i.e., at a mastery level). The updated scoring algorithms are inclusive of emerging communication behaviors that sometimes appear over the past 30 days and provide scores for both overall communication ability and expressive, receptive, and pragmatic forms of communication. Compared to the original Mastery scoring algorithm, the Emerging & Mastery scoring algorithm extends the ORCA measure’s utility in providing reliable measurement for individuals with lower levels of communication ability. In addition, the Emerging & Mastery scoring algorithm has utility in clinical trials that collect repeat assessments in short time intervals when the communication behavior may be emerging but not yet mastered.

Both scoring algorithms had little to no floor or ceiling effects in either AS study; one included children as young as 2 years (original validation study), and the other included children as young as 6 months (AS-NHS). Importantly, there was almost no individual at the highest possible ORCA T-score with the 75th percentile being a little over half a standard deviation above the center of the distribution (3rd quartile range 55.9 to 58.5, Table 4). Thus, if a treatment is provided that improves an individual’s communication abilities, there is evidence that the ORCA measure can reliably estimate variability in communication ability, supporting its use in tracking potential changes over time, as presented by the information functions in Figs. 1 and 2.

A key question for end users is, “Which option for scoring the ORCA measure should I use?” These recommendations are for the AS population and other NDD populations with similar communication abilities. If the outcome (i.e., concept of interest) is overall communication ability and the goal is to have one single score to examine change over time or ability level at a single time point, then either the Mastery or the Emerging & Mastery algorithms perform well. Additionally, our original validation study of the ORCA measure confirmed a single overall dimension of communication inclusive of expressive, receptive, and pragmatic forms of communication [19].

An investigator may select the Mastery scoring algorithm to ensure that any abilities or change in abilities compared to a previous assessment point reflects regular and consistent communication ability over the past 30 days. This algorithm potentially avoids emerging communication behaviors (i.e., “sometimes”) that are not a part of the child’s routine communication. However, if there is an expectation that the study target population will have many individuals at the lower end of communication ability, the Emerging & Mastery scoring algorithm is recommended. If ability levels are unknown, the Emerging & Mastery scoring algorithm is also recommended, as it has a wider spread of reliable measurement across a broader range of ability levels (Fig. 1). Another strength of the Emerging & Mastery scoring algorithm is that it captures behaviors when the individual may use different modalities to achieve the same communication. For example, when greeting a familiar person, the child may “sometimes” use a gesture (e.g., wave hand) and “sometimes” use their device to say hello. This behavior would not be credited under the Mastery scoring algorithm.

If the outcome of interest is one or more forms of communication (i.e., expressive, receptive, pragmatic), then the Emerging & Mastery scoring algorithm is recommended as we found a superior increase in performance in terms of precision compared to the Mastery algorithm for these three forms of communication (Fig. 2). These specific forms may be of interest if an individual is expected to be stronger in one area but not another, or if growth is expected in one area that may subsequently lead to changes in others. For example, the receptive and expressive language domains are considered to be dynamic and interdependent systems. So the strength and relationship between the domains may vary across children and may shift throughout the child’s development [43, 44].

This study has a few limitations. While it was important to confirm the performance of the ORCA scores in both studies, it is highly likely that there was an overlap of participants between each, given AS is a rare disease, and our participants were from the United States and Canada. We do not know the exact time interval between participation in each study for those caregivers who may have participated in both, but we do know that data were collected at least a year apart because the ORCA measure was included in the AS-NHS following the original validation study. There were statistically significant differences between the two samples in caregivers’ education levels, children’s ethnicity and racial groups, and children’s AS genotype. Our findings were based on the U.S. English version of the ORCA measure but should generalize to the language translations of the ORCA measure as the translation company used best practice procedures for the translations [45]. Working in rare disease populations also presents challenges for the statistical approaches used, balancing the value of using a well-established methodology like IRT with the need to make sure the model’s assumptions are met to have confidence in the parameter estimation.

### Conclusion

Our study reflects the need to continue to enhance the ORCA measure and build a body of evidence for its reliability and validity to support its use in AS and other NDD populations with similar levels of communication ability [19–22]. All investigators with licenses for the ORCA measure have access to these scoring algorithms with supporting documentation. This access provides investigators the flexibility to score the ORCA measure based on the concept of interest and study population. We encourage investigators to publish results from using the ORCA measure, noting which scoring algorithm they are using and its justification, or provide the data back to the developers so they may continue to learn about its strengths and limitations. Additional studies are planned to provide more information on interpreting ORCA scores, including estimates for meaningful score differences and meaningful score ranges following best practice guidelines [46]. Given the ongoing advances in gene-targeted therapeutics under study, we must have regulatory-grade measures that reflect what is most important to family caregivers to provide evidence of treatment benefit for their children.

## Data Availability

The datasets analyzed during the current study are not publicly available but may be made available upon reasonable request from the corresponding author.
